# Semi-automated Root Image Analysis (saRIA)

**DOI:** 10.1038/s41598-019-55876-3

**Published:** 2019-12-23

**Authors:** Narendra Narisetti, Michael Henke, Christiane Seiler, Rongli Shi, Astrid Junker, Thomas Altmann, Evgeny Gladilin

**Affiliations:** Molecular Genetics, Leibniz Institute of Plant Genetics and Crop Plant Research (IPK), OT Gatersleben, Corrensstr. 3, Seeland, 06466 Germany

**Keywords:** Image processing, Field trials

## Abstract

Quantitative characterization of root system architecture and its development is important for the assessment of a complete plant phenotype. To enable high-throughput phenotyping of plant roots efficient solutions for automated image analysis are required. Since plants naturally grow in an opaque soil environment, automated analysis of optically heterogeneous and noisy soil-root images represents a challenging task. Here, we present a user-friendly GUI-based tool for semi-automated analysis of soil-root images which allows to perform an efficient image segmentation using a combination of adaptive thresholding and morphological filtering and to derive various quantitative descriptors of the root system architecture including total length, local width, projection area, volume, spatial distribution and orientation. The results of our semi-automated root image segmentation are in good conformity with the reference ground-truth data (mean dice coefficient = 0.82) compared to IJ_Rhizo and GiAroots. Root biomass values calculated with our tool within a few seconds show a high correlation (Pearson coefficient = 0.8) with the results obtained using conventional, pure manual segmentation approaches. Equipped with a number of adjustable parameters and optional correction tools our software is capable of significantly accelerating quantitative analysis and phenotyping of soil-, agar- and washed root images.

## Introduction

Plant roots are key drivers of plant development and growth. They absorb the water and inorganic nutrients from the soil^1–3^ and provide anchoring of the plant body^4,5^. Root system architecture (RSA), the spatial configuration of a root system^1^ is known to be an important phenotypic feature closely related to crop yield variability upon changes in environmental conditions^6,7^. In general, the RSA and its response to the environment are known to be dependent on multiple factors including the plant species, the plant genotype, composition of the soil, availability of nutrients and the environmental conditions^8^. The emerging discipline of plant phenomics aims to extract the plant anatomical and physiological properties to study the plant performance under given conditions^1^. In the case of roots, the relevant traits include descriptors of global and local root morphology (like total length, area, volume, and diameter, or lateral branching, the direction of a tangent, etc.)^9–12^. Monitoring of these traits enables conclusions about the ability of plants to respond to variable environmental factors such as drought, cold, starvation, etc.^13^.

In recent years, a number of approaches to root imaging and image analysis were suggested^14^. However, most of these works rely on the measurement of washed roots or roots grown in artificial, optically transparent media such as liquids or gels^15,16^ that allow a straightforward image analysis. Further non-destructive methods including X-ray computed tomography^17–20^, nuclear magnetic resonance (NMR) microscopy^21^, magnetic resonance imaging^22,23^ and laser scanning^24^ provide unique insights into 3D organization of living root architecture, however, their throughput capabilities are presently rather limited. To enable 2D imaging of roots in a soil-like environment, near-infrared (NIR) imaging of roots growing along surfaces of transparent pots or minirhizotron was designed and tested^25,26^. Special long pass filters were used to block root exposure to visible light and the images were taken by NIR sensitive camera with suitable illumination. The system allows a non-invasive acquisition of root images in darkness^26^.

To analyze a large number of root images in an automated high-throughput manner, a number of software tools are available. Most of these tools were, however, designed to extract RSA traits from specific imaging systems, e.g., images from minirhizotron^25^ and images of roots grown in agar^27^. In addition, some general tools are available for an in-depth analysis of monocot root systems regardless of root structure. These tools depend on significant user input for processing even though they can be used in a batch mode^28–31^. Moreover, some tools can be used as a plugin for general image processing platforms like ImageJ to perform specific tasks for the manual segmentation of roots in the image^32^.

The majority of software for root image analysis is rather tailored to artificial setups such as transparent growing media that cannot be applied to the analysis of heterogeneous and noisy soil-root images. With the exception of software for analysis of X-ray micro-computed tomography (*μ*CT) images^33^ and Root1^34^, which still requires extensive human-computer interaction and suitable for X-ray tomography 3D images. In recently published works^35–37^, novel machine and deep learning approaches to automated segmentation of soil-grown root images were presented. However, the presented approaches rely on color information and require a substantial amount of ground truth training data as well as substantial computational resources.

In this work, we present a GUI-based handy tool for semi-automated root image analysis (saRIA) which enables rapid segmentation of diverse 2D root images including potting soil and artificial media setups in a high-through manner. Based on a combination of adaptive image enhancement, adjustable thresholding and filtering as well as optional manual correction, saRIA represents a broadly applicable tool for quantitative analysis of diverse root image modalities as well as generation of quality ground truth reference images for the training of advanced machine learning/deep learning algorithms.

The paper is structured as follows: Materials and Methods section describes the methodological framework of saRIA including data preparation, segmentation algorithm, and root trait computation. Results section shows the segmentation capabilities of saRIA in comparison to freely available tools and presents the results of roots traits derivation from manually and saRIA segmented root images. In Discussion, we summarize the results of an evaluation study using the saRIA root image segmentation and give an outlook of possible future improvements.

## Methods

Three different modalities for imaging of root system architecture were analyzed in this study including

**Figure 1:**
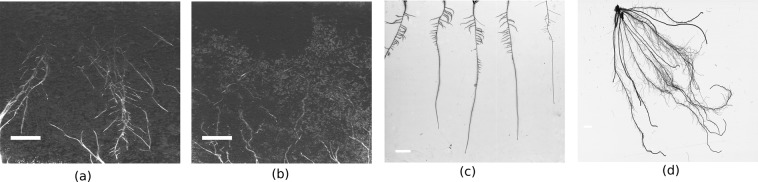
Examples of root images from different setups: (**a**) Arabidopsis plant roots 28 days after sowing, (**b**) Arabidopsis plant roots with condensation noise 28 days after sowing, (**c**) roots grown in agar, (**d**) washed roots. The white color bar on each image represents the scale of 1 cm length.
- **Agar-root image**: During this experiment, the plants were grown on 1/2 MS, 1.5% (w/v) agar medium (pH 5.6 without sugar) in Petri dishes for 5 days. The images were captured by scanning the dishes in grayscale at 300 dots per inch resolution using an Epson Expression 10 000 XL scanner (Seiko Epson)^26^. An exemplary image of Rapeseed roots is shown in Fig. 1c. This image has a clear contrast between roots and homogeneous background. Nevertheless, the background pixels have some morphological artifacts which will be discussed in the next subsection.
- **Washed-roots image**: Maize plants were grown in transparent pots filled with a mixture of substrates (self-made compost, IPK) and sand (1:1) for 3 weeks. This digital image is obtained by scanning the washed maize roots on an Epson Expression 10 000 XL scanner (Seiko Epson) as shown in Fig. 1d. Compared to the above two types of root images, it is less noisy and the contrast between roots and background is significantly higher.

- **Soil-root image**: This type of digital image is taken by a monochrome camera (UI-5490SE-M-GL, IDS) with LED illumination (UV, 380 nm) in a custom-made imaging box similar to our previously published setup^26^. In brief, plants are grown in transparent pots [77 × 77 × 97 mm (WxLxH))] filled with the potting substrate (Potgrond P, Klasmann). An example of soil-root images acquired with this system is shown in Fig. 1a. Depending on the developmental stage, plant health, environmental factors (e.g., temperature, humidity), these images may, in general, exhibit diverse artifacts including low contrast between the root architecture and heterogeneous soil, inhomogeneous scene illumination (i.e. vertical intensity gradient), water condensation at the pot walls, see Fig. 1b. Identification of relevant root architecture in such structurally and statistically noisy images represents a challenging task.

**Figure 2:**
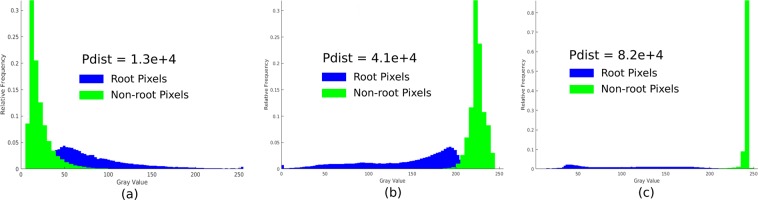
Histograms of root (blue) and non-root (green) intensity values of different imaging modalities shown in Fig. 1: (**a**) soil-root, (**b**) agar grown roots and (**c**) washed roots images. The pairwise distance (Pdist) between root and non-root histograms, which serves as a quantitative measure for separability of root and non-root image structures, indicates that soil-root images represent the most challenging modality for image segmentation.

Consequently, the classification between the root and non-root pixels in these three image modalities is unequally difficult. Figure 2 shows histograms and pairwise distances (Pdist = pdist2(A, B)) between root and non-root grayscale distributions corresponding to soil-root, agar and washed roots images in Fig. 1, respectively. As expected, soil-root images exhibit a strong overlap between grayscale values of root and non-root pixels and the distance between them is the lowest among these three imaging modalities. Therefore, here we focused on soil-root image analysis only. Application of our approach to higher contrast image modalities (i.e. agar, washed roots images) is, however, trivial, and only requires inverting the grayscale values.

**Figure 3:**
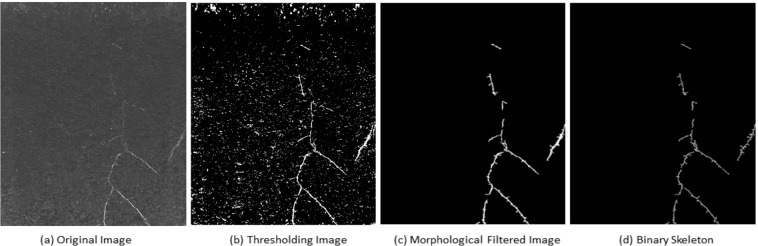
Basic steps of root image processing and analysis in saRIA: (**a**) Arabidopsis soil-root image, (**b**) adaptive thresholding, (**c**) morphological filtering, (**d**) root skeleton.

### Image analysis

**Figure 4:**
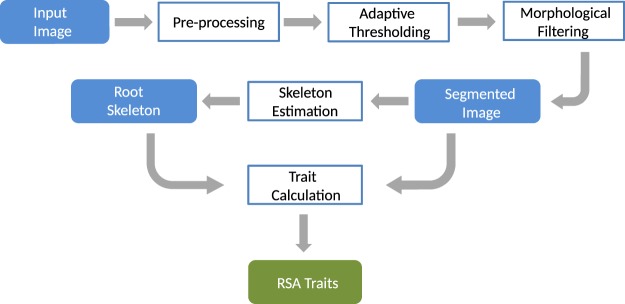
Work flow of image processing and analysis in saRIA. Color filled icons indicate the data modalities, framed rectangles describe image processing steps.
- **Evaluation**. To examine the performance of image segmentation, a standard statistical metric, the Dice similarity coefficient (DSC)^40^, is used. The DSC evaluates the spatial overlap between two binary images and its value ranges between 0 (no overlap) to 1 (perfect overlap). The DSC is defined as follows:

Image analysis algorithms, as well as the graphic user interface (GUI) were implemented under the MATLAB 2018b environment^38^. The major goal of image analysis consists of segmentation of root architecture and calculation of phenotypic features of root architecture and image intensity (i.e. color). In the case of color images, the input image is converted to a grayscale image using the *rgb2gray* Matlab routine. In general, the pipeline of image analysis includes the following steps:

- **Image I/O**. Most standard image formats (such as *.jpg, *.png, *.bmp, *.tif) can be imported for further processing and analysis. Stepwise single image, as well as automated processing of large image datasets is implemented.
- **Image preprocessing**. Depending on the imaging modality (e.g., soil-root or agar-root images) and the presence of noisy or structural artifacts, preprocessing steps may include cropping of the region of interest (ROI), inverting of image intensity, despeckling and smoothing. In the case of agar and washed-root images, inversion of image intensity has to be performed prior to image analysis. Otherwise, the procedure of agar and washed-root image analysis is similar to soil-root images.
- **Adaptive image thresholding**. Preprocessed images are segmented into a foreground (roots) and background using adaptive thresholding based on Gaussian weighted mean as suggested by^39^. This technique tolerates global inhomogeneity of image intensity such as vertical image gradient in our soil-root images. An example of an adaptive thresholding of Arabidopsis soil-root image (Fig. 3a) is shown in Fig. 3b.
- **Morphological filtering**. To remove white noise and small non-root blob-like structures (such as sand, gravel or water condensation artifact in Fig. 1b) morphological filtering is applied. Thereby, roots are considered to be elongated line-/curve-like structures that differ from this kind of non-root blobs with respect to their area, length, and shape (i.e. eccentricity). In the case the root region represents a single connected structure, filtering can be performed merely by applying intensity and area thresholds. If roots are represented by disconnected structures, differentiation of root from non-root structures is performed using additional shape descriptors such as length and eccentricity, i.e. a descriptor of the object’s Eigenellipse elongation, which is zero for an absolutely round and 1 for a line-object. By appropriate setting of thresholds for these three parameters, non-root blobby structures are removed. Figure 3c shows an example of morphological filtering of a preliminary segmented root image.
- **Skeletonization**. Root skeleton is calculated on the basis of the segmented and filtered image. In addition to the filtering steps described above, additional thinning or eroding of the binary image is applied to suppress high-frequency noise. The exemplary image for the skeleton extraction is shown in Fig. 3d.
- **Root feature calculation**. The distance transform of the cleaned binary image is calculated for assessment of the local root width (or diameter) measured in pixels of the root skeleton. Further root features include root length, root angles with respect to a vertical axis, branching and end points of the root skeleton, the intensity of root pixels and their standard statistical descriptors (i.e. mean, stdev values). The complete list of a total of 44 root traits can be found in Supplementary Information (Table S1). Note that all traits are extracted using pixel-wise calculation irrespective of a number of root systems in the image. In addition, the RSA traits can also be written out in mm. For this purpose, the user has to set the pixel-to-mm conversion factor in the saRIA GUI. Here, we derived the pixel-to-mm factor by measuring a reference line (white color bar) in the images as shown in Fig. 1. The pixel-to-mm conversion factor (CF) is then defined as follows:
  1 $${\bf{CF}}=\frac{length\,of\,there\,ference\,line\,in\,mm}{length\,of\,there\,ferenc\,eline\,in\,pixels}$$
  The workflow of image analysis is also shown in Fig. 4.

2 $$DSC=\frac{2\ast TP}{2\ast TP+FP+FN},$$

where TP, FP, and FN are true positive, false positive and false negative pixels, respectively.

## Results

### Semi-automated segmentation of soil-root images

To evaluate the performance of our algorithms, segmentation of 100 Arabidopsis soil-root images was performed automatically and compared with the results of fully manual segmentation. Thereby, manual segmentation was also carried out with saRIA by applying a low-intensity threshold for selection of all high- as well as low-intensity roots, and subsequently followed by manual removal of all remaining artifacts including solitary objects as well as noise regions attached to the root (which cannot be otherwise identified and quantified as a separate object) using the ‘clearInside’ saRIA tool. This step was done by two biologists (co-authors of this manuscript) with expertise in RSA. In contrast, for semi-automated image segmentation user merely has to adjust the four basic algorithmic parameters (controlled by the four GUI sliders) according to the subjectively best result of visual inspection of a few test images. Once the best combination of algorithmic parameters is defined, segmentation of all remaining images can be performed in a fully unsupervised manner.

**Figure 5:**
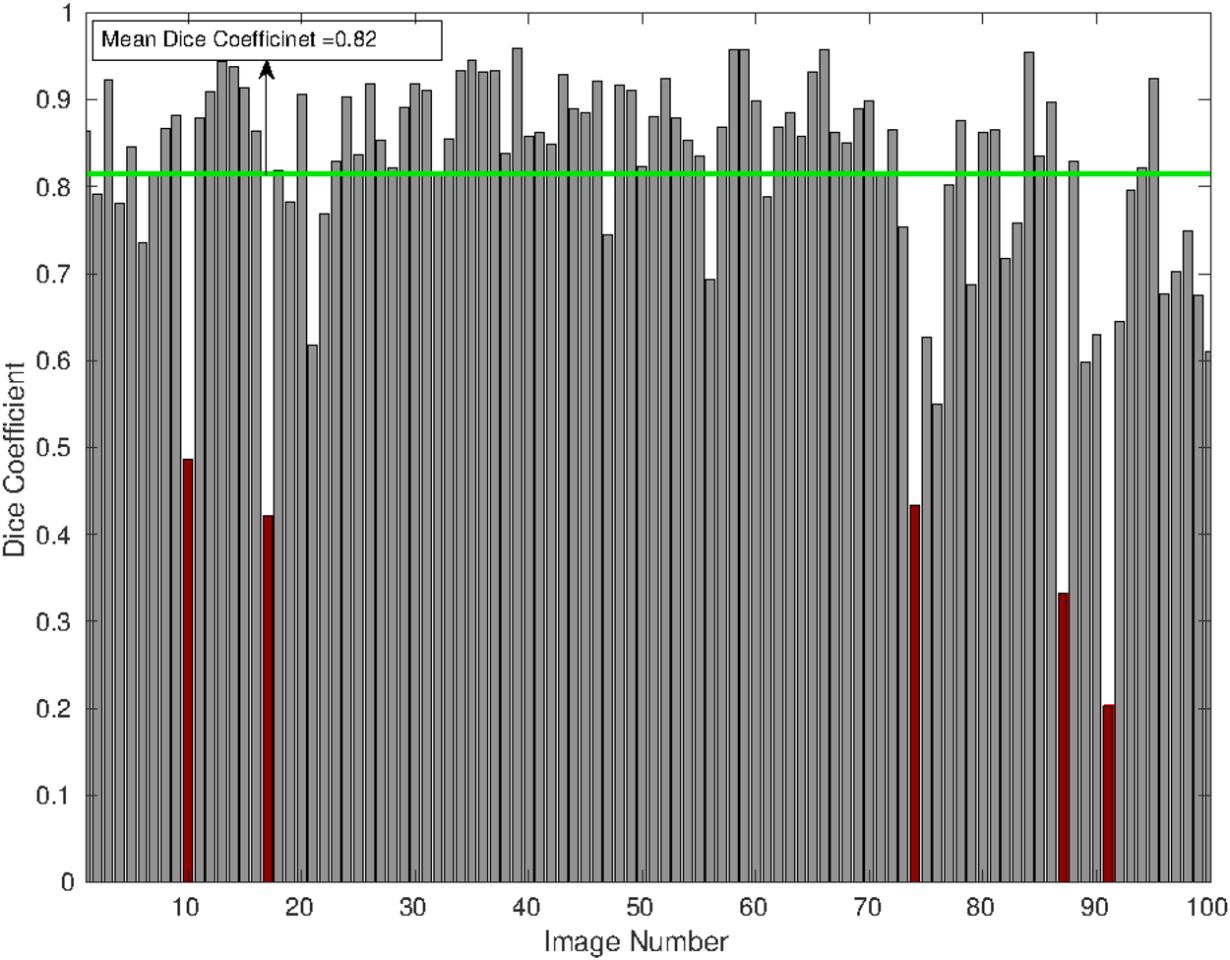
The accuracy of root image detection using saRIA vs. manual segmentation (ground-truth data) in terms of the Dice similarity coefficient (DSC). The green line points to the mean DSC value over 100 tested soil-root images. The red color bars indicate few cases of poor saRIA performance with a low DSC value that corresponds to roots in the early stage of plant development.

Figure 5 shows the performance of image segmentation compared to manual segmentation of 100 soil-plant images for the intensity threshold (T) 0.12, minimum area 450, minimum length 46 and minimum eccentricity 0.49. This figure shows that approximately 90% of images have DSC values greater than 0.7 and the mean DSC value is 0.82.

To validate the robustness of the saRIA image segmentation of the above 100 images, we have also compared with two other freely available tools called IJ_Rhizo^41^ and GiA roots^42^. Table 1 shows the mean DSC value for the subjectively best possible configuration of IJ_Rhizo, GiAroots with three different thresholding methods and saRIA. The table briefs that saRIA significantly outperformed with the combination of Gaussian adaptive thresholding and all morphological parameters (area, length, circularity) compared to the IJ_Rhizo and GiAroots. The brief discussion on parameter configuration of IJ_Rhizo and GiaRoots can be found at Pierret *et al*.^41^ and Galkovskyi *et al*.^42^ respectively.

**Table 1:** Comparison of saRIA image segmentation quality (i.e. mean DSC) and parameters with IJ_Rhizo and GiAroots.

Parameter	IJ_Rhizo	GiAroots-Global threshold	GiAroots-Adaptive threshold	GiAroots-Double adaptive threshold	saRIA
Mean DSC	0.43	0.50	0.54	0.69	0.82
Threshold	T1 = 50, T2 = 255	T = 50	Mean shift = −2.0096	Bound drop value = 5	T = 0.12
Minimum area	5 mm	450	450	450	450
Minimum length	x	x	x	x	46
Circularity	0.7 (0 - line, 1 - circle)	x	x	x	0.49 (0 - circle, 1 - line)

### Evaluation of phenotypic traits vs. smartroot

**Figure 6:**
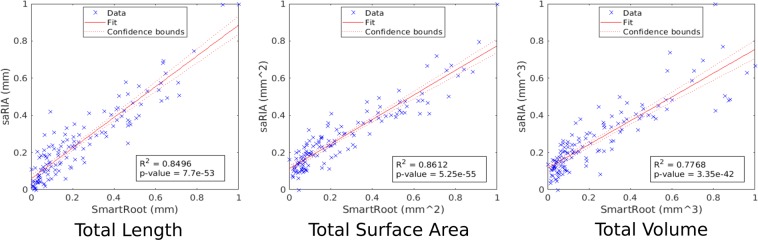
Correlation between root traits calculated using semi-automated saRIA (y-axis) and manual SmartRoot (x-axis) image segmentation. Each point represents a trait value estimated from one of 126 soil-root images. The red color solid line and dotted lines represent a fitted curve and 95% confidence bounds, respectively. The *R*^2^ value indicates good conformity between saRIA and SmartRoot results of image segmentation and trait calculation.

Here, the results of phenotypic root characterization obtained with saRIA are evaluated in comparison with SmartRoot^32^. The SmartRoot is the most widely used for the traits quantification of disconnected RSA and each part of the root is traced manually by placing multiple landmarks finally interconnected to the root skeleton. However, the SmartRoot doesn’t deliver the single-segmented binary (reference) image for the comparison with saRIA. Therefore, the root traits derived from such manually segmented images can be seen as a reference ground-truth data. To quantify the (dis)similarity between saRIA and SmartRoot results, the correlation coefficient of determination *R*^2^ and significance level p-value are used. They represent the percent of the saRIA calculated traits that are closest to the ground-truth data and model validation respectively. Figure 6 shows the correlation between the SmartRoot (x-axis) and saRIA (y-axis) outputs for three traits where each point denotes one particular image out of 126 Arabidopsis root images acquired with our in-house soil-root imaging system. Note that the images used for traits evaluation are different from the above segmentation evaluation data. The three traits used for evaluation are the total root length, total root surface area, and total root volume. As one can see, for all three traits correlation between saRIA semi-automatically and SmartRoot manually segmented images exhibit *R*^2^ values higher than 0.84, 0.86, 0.77 and p-values 7.7e-53, 5.25e-55, 3.35e-42 respectively.

### Visualization of root features

**Figure 7:**
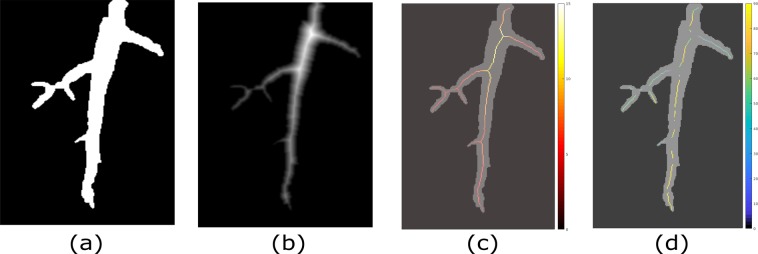
Visualization of root features: (**a**) binary root image, (**b**) corresponding Euclidean distance map, (**c**) overlay of the binary image with the root skeleton colored according to the local root width (gray-scale map), (**d**) overlay of the binary image with the root skeleton colored according to the local root tangent (black-yellow color map).

In addition to numerical outputs, saRIA software generates root features (e.g., distance maps, skeletons, width distributions, etc) for visualization purposes. Figure 7 shows an example of images of root width and orientation. The root width is calculated as the Euclidean distance between root skeleton and root boundary pixels. Figure 7b depicts the Euclidean distance map of root object Fig. 7a where high-intensity central pixels represent the root width. The corresponding width color map is shown in Fig. 7c. The width feature is useful to calculate the root volume and surface area for biomass estimation.

Figure 7d displays the absolute orientation of each root skeleton pixel with respect to the horizontal axis in black-yellow color-map representation. Here, a validated linear regression model was used to calculate the slope of a pixel in the skeleton image. The local slope in *i*-th pixel is obtained by fitting a tangent line to the fraction of root skeleton framed by a 15 × 15 pixel mask around the *i*-th pixel. An exemplary figure for the local linear fit can be found in Supplementary Information (Fig. S1). The validated regression model means that only pixels satisfying the regression model with a high confidence level (i.e. p-value < 0.05 and *R*^2^ > 0.5) were accepted. Pixels with low confidence of the linear fit model such as root branches with the non-linear distribution of skeleton pixels were excluded by the calculation of global statistics of root orientation.

## Discussion

The objectives of our GUI-based saRIA tool are to automatize the time-consuming manual segmentation of structurally complex and noisy root images and to enable calculation of RSA traits from different root imaging modalities including soil, agar and washed roots images. Using this approach, root architecture can be rapidly segmented and quantified by adjusting a small set of algorithmic parameters. Segmentation with saRIA is particularly efficient when background structures differ from roots in geometrical parameters (such as shape and size) and grayscale intensity. Artifacts resembling optical root appearance, e.g., scratches on the pot surface or high-intensity areas resulting from water condensation, are, in contrast, difficult to eliminate in a fully automated manner. Such artifacts can, however, be removed using a manual segmentation tool also available with saRIA.

The accuracy of trait estimation in saRIA depends on the quality of semi-automated image segmentation. Our solution for analyzing a large number of images is to define the best possible set of algorithmic parameters for a subset of representative root images and then to apply this configuration to all remaining images in a fully automated manner. Here, 15% of input data with different scenarios (low, medium and high dense root images) are considered for the best possible configuration settings.

The quality of image segmentation from Table 1 explains that the global thresholding methods in IJ_Rhizo (bi-level threshold) and GiAroots (single-level threshold) under-performed than adaptive thresholding methods. Since the global thresholding methods contain one or two threshold values for a complete image that preserve the high-intensity noisy objects and removes the low-intensity roots in the soil-root image. However, the GiAroots also implemented based on adaptive thresholding but it lacks the Gaussian smoothing filter in the preprocessing step and morphological constraints (i.e. length and circularity) on binary root objects, represented as x in Table 1. Therefore, the combination of adaptive thresholding and morphological filtering promising more accurate segmentation in saRIA for soil-root images.

**Figure 8:**
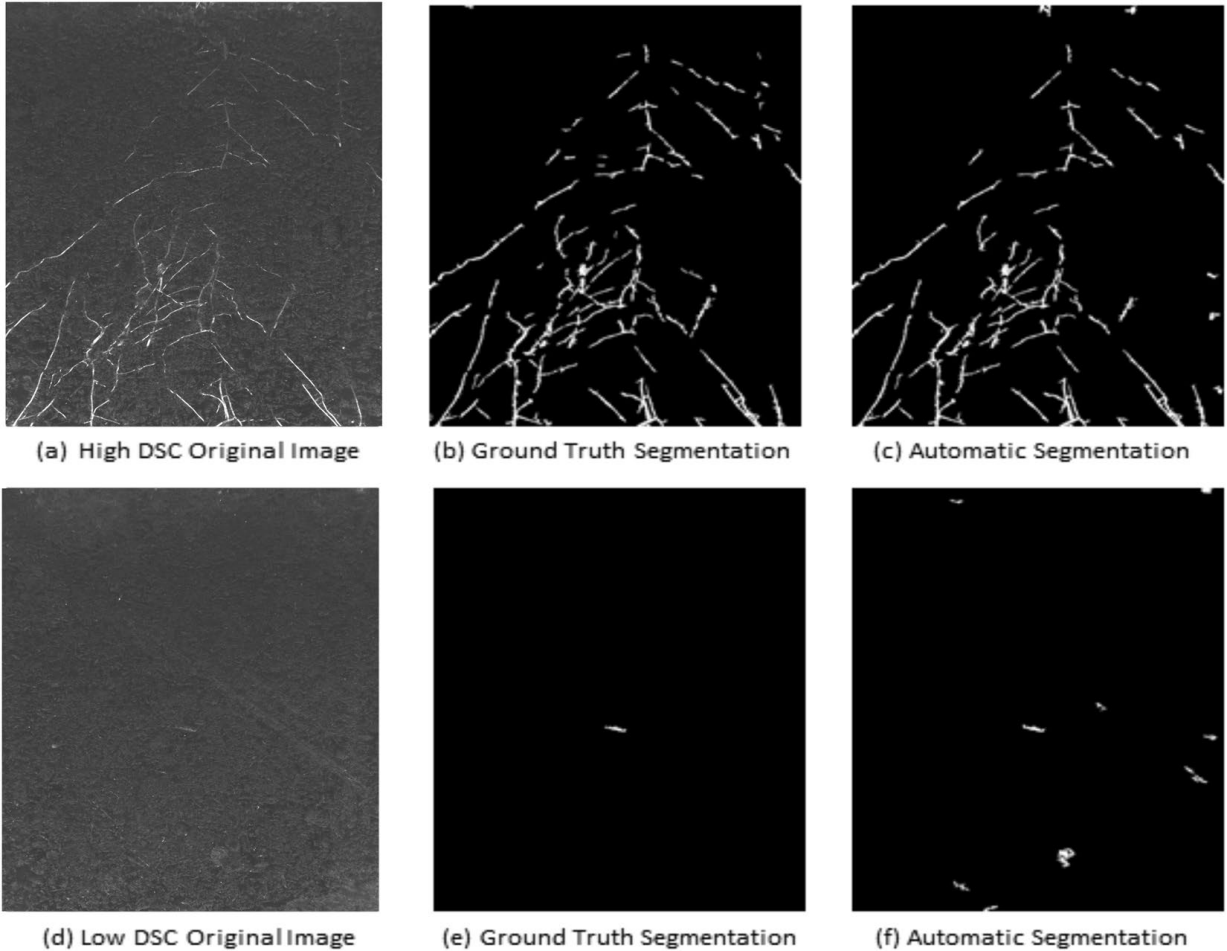
Comparison of root image segmentation for young/small vs. large/adult plants. Top row shows (**a**) original, (**b**) ground-truth and high-DSC saRIA-segmented images of a large root, bottom row (**d**–**f**) shows an example of a relatively small root at the early stage of Arabidopsis plant development with a low DSC corresponding to the red bar number 91 in Fig. 5.

From the summary of our automated image analysis in Fig. 5, it is evident that the accuracy of root image segmentation is tendentially higher (DSC > 0.9) for images with large root architecture. Figure 8 shows example images of small and large root architecture that exhibit low and high DSC of automated segmentation vs. ground-truth, respectively. Because the large root architecture requires a low value for threshold and high value for morphological parameters compared to the small root architecture image where most of the disconnected root components are small in morphology. It results in the removal of small-sized roots and keeping the (both disconnected root and heterogeneous soil) structures that are big in morphology. This observation confirms that the relative error in the segmentation of small root architecture (see red color bars in Fig. 5) from background pixels is higher for automated segmentation in comparison to the large roots. However, these artifacts can be overcome by setting low values for morphological parameters in the segmentation configuration.

The quantitative comparison of saRIA is limited to SmartRoot because other software solutions for root image analysis are either tailored to a non-interrupted representation of root architecture like RootNav^43^ or restricted to high contrast imaging modalities, e.g., agar grown or washed roots, like GiARoots^42^ that is no longer under development and closed source and not promising more accurate segmentation as shown in Table 1.

As mentioned earlier, saRIA is capable of calculating 44 number of root traits. In brief, they are categorized into 11 features named area (number of root pixels), number of disconnected root objects, total length, surface area, volume, number of branching and ending points, statistical distribution (mean, median, standard deviation, skewness, kurtosis and percentile) of root geometry in horizontal and vertical direction, width and orientation. Among those, three important features for root biomass calculation are presented for saRIA traits evaluation.

The results of our evaluation tests in Fig. 6 show that root traits obtained using saRIA are highly correlating (*R*^2^ > 0.8) and significant (*p*-*value* < 0.05) with manual segmentation in SmartRoot software. Consequently, one can perform comparatively high-quality root phenotyping with saRIA 20 or more times faster than with manual annotation of root structures pixel by pixel.

The difference in trait estimates between saRIA and SmartRoot might result from the image segmentation parameters and root thickness. First, the morphological parameters remove tiny roots which have small area and length. Second, the local thickness of roots in saRIA is defined as the average diameter of automatically segmented roots which include high-intensity structures originating from tiny root hairs that can be avoided in manual segmentation. This may lead to differences in average root width, length and volume assessed with saRIA vs. SmartRoot. However, the ability to interactively adjust parameters is available in the saRIA to improve the trait extraction and even to produce a set of alternative image segmentation in an automated manner, i.e. automated root tracing and trait extraction for the selected configuration.

**Figure 9:**
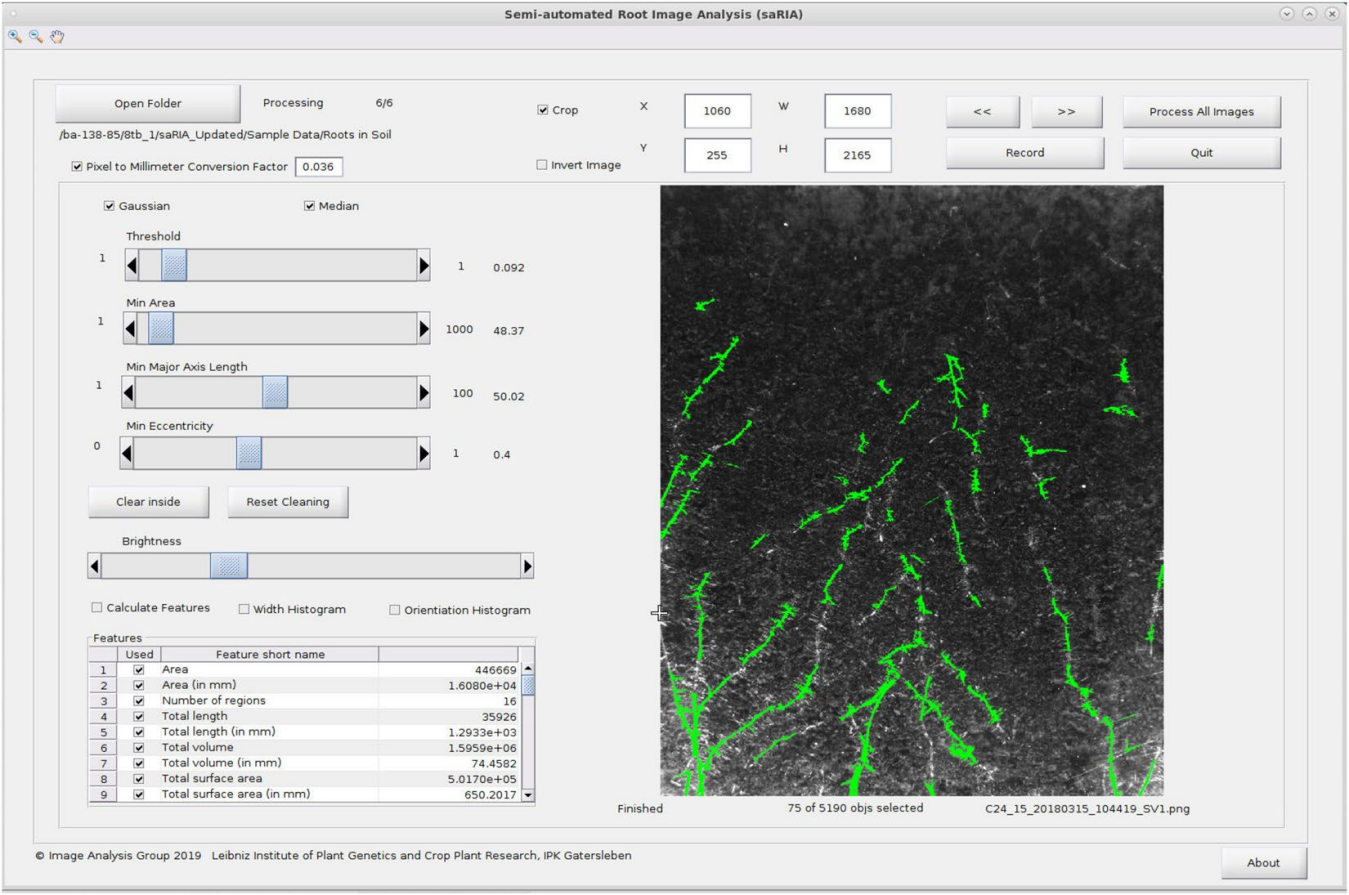
The graphical user interface of saRIA. Green colored pixels indicate segmented root regions of the soil-root image.

Here, we present a user-friendly GUI-based software solution for high-throughput analysis of root images of different image modalities, including challenging soil-root images. Figure 9 shows the GUI of saRIA software which is freely available as a precompiled executable program from https://ag-ba.ipk-gatersleben.de/saria.html. Further examples of agar and washed roots image segmentation using saRIA are included in the Supplementary Information, see Figs. S2 and S3. The saRIA software can be applied for the analysis of single images or large image datasets to automatically detect and extract multiple root traits. This software is designed for end-users with limited technical knowledge to enable them a widely automated analysis of complex soil-root images in an intuitive and transparent manner. The saRIA segmentation, root tracing and trait calculation algorithms require, in average, 5 seconds to process and analyze a 6-megapixel (cropped) image (on Intel(R) Core(TM) i5-4570S CPU @ 2.90 GHz, 64-bit quadcore processor with 4 GB ram and 500 GB HDD) which is significantly faster in comparison to conventional manual segmentation, e.g., SmartRoot. Table 2 summarizes the essential features of saRIA vs. other well-established root image analysis tools (SmartRoot, EZ-RHIZO, and WinRHIZO). The major difference between the saRIA and other available tools (Plant Root - roots grown in cloth substrate in custom rhizoboxes, RootReader2D - need high contrast images and RootNav - supports nested root architectures) is that it is capable of segmenting contrast varying disconnected root architectures (in a semi-automated manner with automated trait extraction) in both potting soil and artificial growing media.

**Table 2:** The feature comparison of saRIA with other softwares for root segmentation and trait extraction.

Feature	saRIA	SmartRoot	EZ-RHIZO	WinRHIZO
License	Free, Closed source	Free, Open source	Free, Closed source	Commercial
Platform	Linux, Windows	Cross-Platform, it is an ImageJ Plugin	Windows only	Windows only
Language	MATLAB	Java	C++	x
Root Tracing	Semi-automated	Manual	Manual and Automated	Automated
Medium	Soil, Agar, Washed	Soil, Agar, Washed	Agar	Washed roots, Agar
Database Support	No, but has CSV export support	SQL	SQL	No, data files are saved in ASCII text format

In addition to routine analysis of root images, saRIA can be used for the rapid generation of ground-truth segmentation data that are highly demanded for advanced machine learning/deep learning techniques.

The study of plant genotype with root phenotype requires a contribution of many groups and utilization of molecular, physiological and imaging techniques. In addition, the performance of phenotype analysis depends on image quality. The segmentation algorithm currently bundled in saRIA is based on the intensity gradient among the pixels. Further extensions of the saRIA segmentation pipeline including advanced machine learning approaches and additional static and dynamic RSA traits like topological data (number of primary and lateral roots, branching angles, lateral density) are planned in the future.

## Supplementary information

Supplementary Information 1

Supplementary Information 2
