# Supplementary material for: Semi-automated Root Image Analysis (saRIA)
Source: Sci Rep. 2019 Dec 23;9:19674. doi: 10.1038/s41598-019-55876-3 (PMC6928233; doi:10.1038/s41598-019-55876-3)

**Supplementary Material**

*Semi-automated Root Image Analysis (saRIA),* N. Narisetti, M. Henke, C. Seiler, R. Shi, A. Junker, T. Altmann, E. Gladilin*.

**Table S1. Root Traits:** The description of estimated root system architecture traits in saRIA software.

| **_Trait Name_** | **_Description_** |
| --- | --- |
| Area | Number of root pixels in the image |
| Number of Regions | Number of disconnected root objects in the image |
| Total Length | The sum of major axis length of each root object  approximated by fitting ellipse to the root object |
| Total Volume (V) | The sum of local volume at each root object of skeleton approximated by tubular shape whose average radius is estimated from image  $V=\sum_{i=0}^{n} \pi{r_{i}}^{2}$  Where $r_{i}$ is the average radius of $i^{th}$ root component in the image. |
| Total Surface Area (SA) | The sum of surface area at each root object of skeleton approximated by tubular shape whose average radius is estimated from image  $SA=\sum_{i=0}^{n} 2\pi r_{i}$  Where $r_{i}$ is the average radius of $i^{th}$ root component in the image. |
| Specific Root Length | The ratio of total length and total volume of roots in the image. |
| Number of Branching Points | The total number of branches in the root skeleton |
| Number of End Points | The total number of end points in the root skeleton |
| Geometrical X_mean, Y_mean | The mean value of root pixels distribution in horizontal and vertical direction |
| Geometrical X_median, Y_median | The median value of root pixels distribution in horizontal and vertical direction |
| Geometrical X_std, Y_std | The standard deviation of root pixels distribution in horizontal and vertical direction |
| Geometrical X_skew, Y_skew | The skewness of root pixels distribution in horizontal and vertical direction |
| Geometrical X_kurt, Y_kurt | The kurtosis of root pixels distribution in horizontal and vertical direction |
| Geometrical X_p25, Y_p25 | The 25 percentile of root pixels distribution in horizontal and vertical direction |
| Geometrical X_p50, Y_p50 | The 50 percentile of root pixels distribution in horizontal and vertical direction |
| Geometrical X_p75, Y_p75 | The 75 percentile of root pixels distribution in horizontal and vertical direction |
| Geometrical X_p99, Y_99 | The 75 percentile of root pixels distribution in horizontal and vertical direction |
| Width mean | Average root diameter |
| Width median | Median root diameter |
| Width std | Standard deviation of the root diameter |
| Width skew | Skewness of root diameter |
| Width kurt | Kurtosis of root diameter |
| Width p25 | 25 percentile of root diameter |
| Width p50 | 50 percentile of root diameter |
| Width p75 | 75 percentile of root diameter |
| Width p99 | 99 percentile of root diameter |
| Orientation mean | Average root Orientation |
| Orientation median | Median root Orientation |
| Orientation std | Standard deviation of the root Orientation |
| Orientation skew | Skewness of root Orientation |
| Orientation kurt | Kurtosis of root Orientation |
| Orientation p25 | 25 percentile of root Orientation |
| Orientation p50 | 50 percentile of root Orientation |
| Orientation p75 | 75 percentile of root Orientation |
| Orientation p99 | 99 percentile of root Orientation |

**Table S2. Data set:** Data used for traits comparison of saRIA (automatic) Vs Smart root (manual) (See attached excel spread sheet to the supplementary information)

**Figure S1: Linear regression fit:** An exemplary analysis of linear regression fit with 10 neighbor pixels. Where β_i_ represents the measured angle between the central pixel (green color blob) and a fitted tangent (gray dotted line).

**
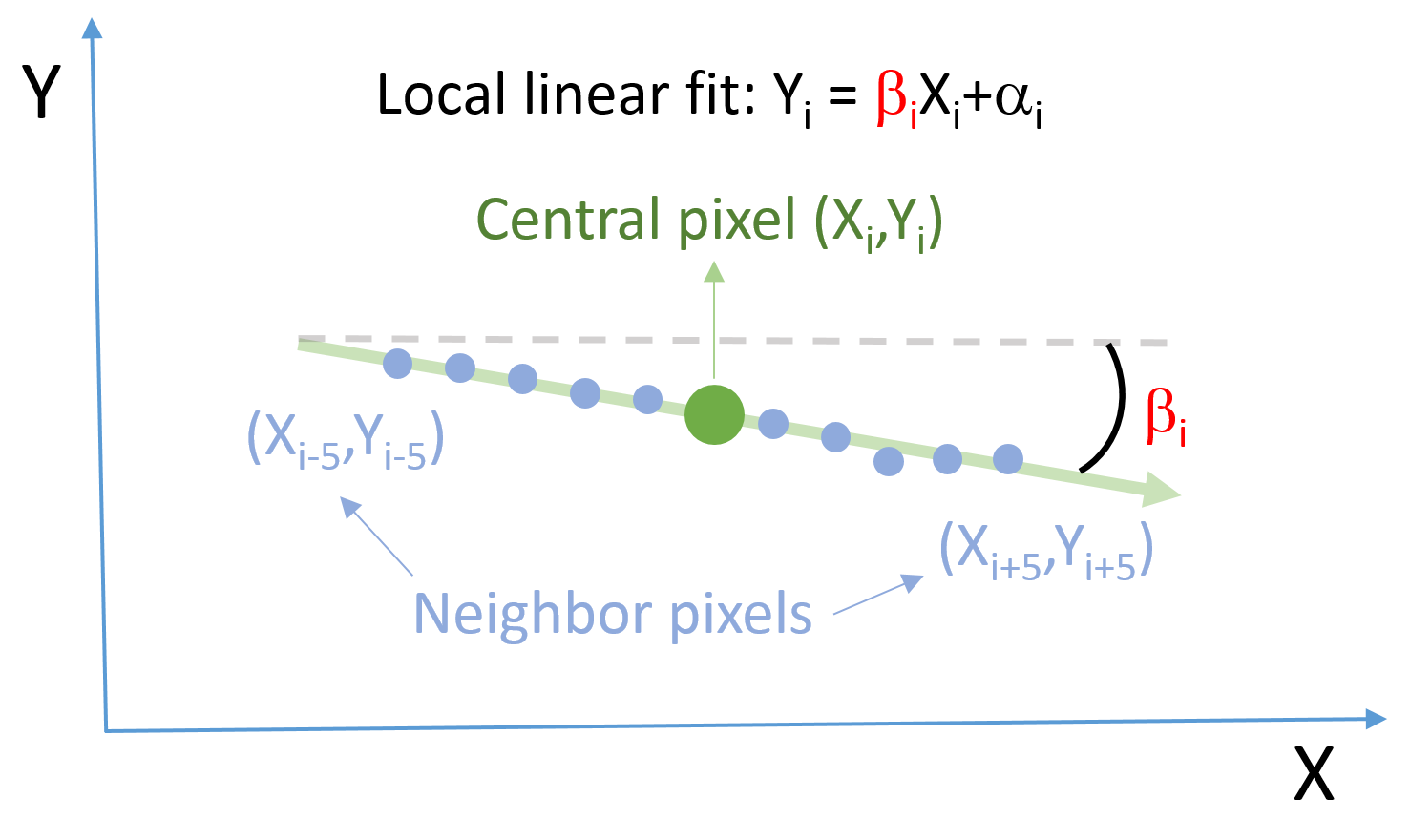
**

**Figure S2: saRIA software:** The graphical user interface of saRIA software. Green colour pixels represents the detected roots in the Agar root image.


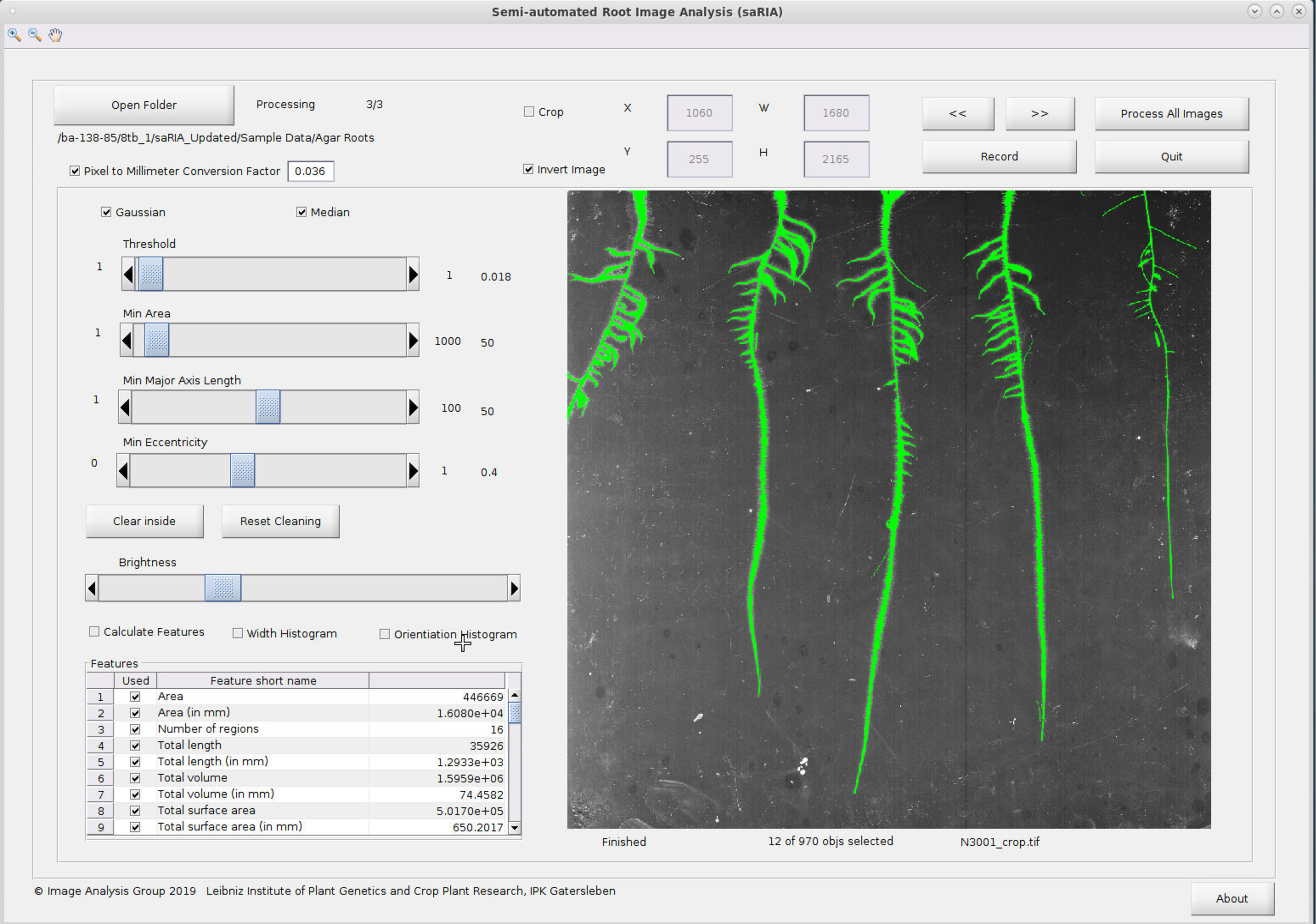


**Figure S3: saRIA software:** The graphical user interface of saRIA software. Green colour pixels represents the detected roots in the scanned image.


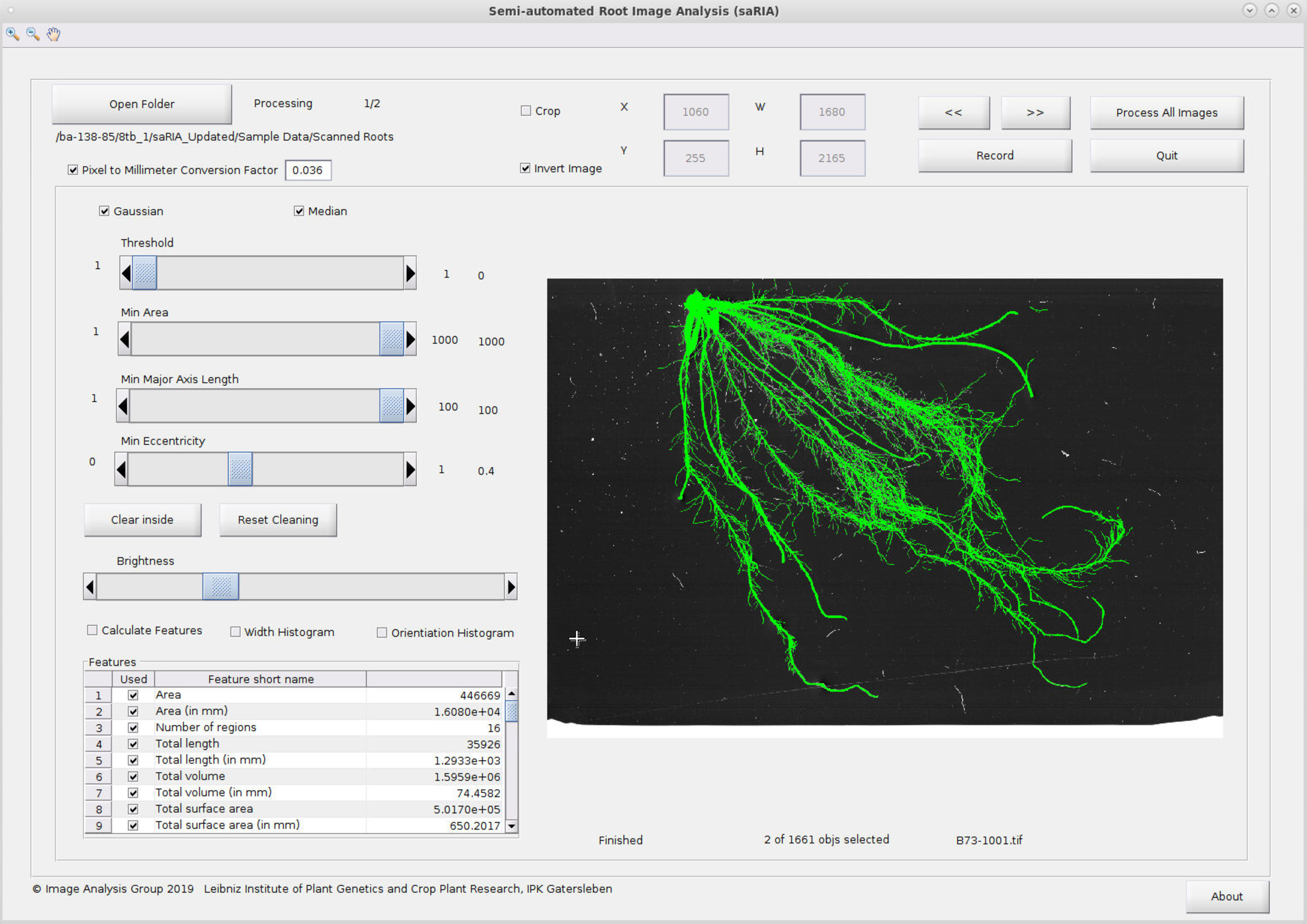

Supplement: Supplementary file 1 — Supplementary Information 1 [file 41598_2019_55876_MOESM1_ESM.docx]
